# Total Hip Arthroplasty: Minimal Clinically Important Difference and Patient Acceptable Symptom State for the Forgotten Joint Score 12

**DOI:** 10.3390/ijerph18052267

**Published:** 2021-02-25

**Authors:** Umile Giuseppe Longo, Sergio De Salvatore, Ilaria Piergentili, Anna Indiveri, Calogero Di Naro, Giulia Santamaria, Anna Marchetti, Maria Grazia De Marinis, Vincenzo Denaro

**Affiliations:** 1 Department of Orthopaedic and Trauma Surgery, Campus Bio-Medico University, Via Alvaro del Portillo 200, 00128 Rome, Italy; s.desalvatore@unicampus.it (S.D.S.); ilaria.piergentili94@gmail.com (I.P.); c.dinaro@unicampus.it (C.D.N.); denaro@unicampus.it (V.D.); 2 Research Unit Nursing Science, Campus Bio-Medico di Roma University, 00128 Rome, Italy; a.indiveri@unicampus.it (A.I.); g.santamaria@unicampus.it (G.S.); a.marchetti@unicampus.it (A.M.); m.demarinis@unicampus.it (M.G.D.M.)

**Keywords:** total hip arthroplasty, forgotten joint score, minimal clinically important difference (MCID), patient acceptable symptom state, hip replacement

## Abstract

The Forgotten Joint Score-12 (FJS-12) is a valid patient-reported outcome measures (PROMs) used to assess prosthesis awareness during daily activities after total hip arthroplasty (THA). The minimum clinically important difference (MCID) can be defined as the smallest change or difference that is evaluated as beneficial and could change the patient’s clinical management. The patient acceptable symptom state (PASS) is considered the minimum PROMs cut-off value that corresponds to a patient’s satisfactory state of health. Despite the validity and reliability of the FJS-12 having been already demonstrated, the MCID and the PASS of this score have not previously been defined. Patients undergoing THA from January 2019 to October 2019 were assessed pre-operatively and six months post-surgery using the FJS-12, the Western Ontario and McMaster Universities Osteoarthritis Index (WOMAC) and the Oxford Hip Score (OHS). Pre-operative and follow-up questionnaires were completed by 50 patients. Both distribution-based approaches and anchor approaches were used to estimate MCID. The aim of this paper was to assess the MCID and PASS values of FJS-12 after total hip replacement. The FJS-12 MCID from baseline to 6 months post-operative follow-up was 17.5. The PASS calculated ranged from 69.8 to 91.7.

## 1. Introduction

There are several types of measurement tools used in orthopedics, including subjective (tests and scores) and objective (laboratory tests) parameters. Patient-reported outcome measures (PROMs) are commonly used in orthopedic studies to assess patient symptoms and health after surgical treatment [1]. PROMs are self-reported measures, designed with the aim of collecting information related to constructs that are reported by the patients themselves, without third party interpretations. PROMs include perception of pain, functionality, prosthesis awareness, satisfaction and health-related quality of life [2,3]. PROMs can be generic, including health-related information, or specific, reporting data on specific diagnosis or procedures (joint replacement, surgery or a specific diagnosis) [4].

The Forgotten Joint Score (FJS-12), the Western Ontario and McMaster Universities Osteoarthritis Index (WOMAC) and the Oxford Hip Score (OHS) are examples of PROMs [5]. The FJS-12 is a patient-reported outcome scale created to evaluate prosthesis awareness during daily activities [6]. Developed by Behrend et al. in 2012, it assesses the prosthetic joint’s degree of awareness, with a low ceiling effect [6].

Total hip arthroplasty (THA) has revolutionized the treatment of hip arthritis. This surgery is related to a high number of risk factors and complications. Bone cement implantation syndrome [7], hip fractures [8] and residual lateral hip pain [9] could affect patients during and after THA surgery. The complications could influence the outcomes [10], the quality of life and the functionality of the joint [8]. The number of THAs performed worldwide increases every year [11,12]. Therefore, finding valid solutions to assess the patient’s outcomes after surgery is mandatory. The efficacy of a surgical procedure is usually evaluated by the mean difference between pre- and post-operative scores. Although this difference could measure a change in outcomes after THA, it does not reflect the effect size [13]. A significant mean change (expressed by significant *p*-value), could not be transposed into a considerable change for the patient [14]. Moreover, the clinical significance for individual patients may vary widely; therefore, it is necessary to find and evaluate new scores that are as realistic as possible. Remarkable changes in scores can be defined by the minimum clinically important difference (MCID) [15]. The term MCID is always interchangeably used with minimum important change (MIC) [16], causing ambiguity [14]. The MCID can be defined as the smallest change or difference (assessed by PROMs) that is evaluated as beneficial and could change the patient’s clinical management, assuming a lack of significant side effects and costs [17]. The difference in the mean change of FJS-12 between patients without improvements compared to patients with “small” improvements after a THA could be defined as MCID. Another significant value in the literature is the patient acceptable symptom state (PASS). It is considered as the minimum PROMs cut-off value that corresponds to a patient’s satisfactory state [18]. The PASS is the threshold for PROMs most closely associated with patient satisfaction, and is assessed on a separate questionnaire (the anchor) [14]. Despite the validity and reliability of the FJS-12 being already demonstrated [19], the MCID and the PASS of this score have not previously been assessed.

This study aimed to define the MCID and PASS for the FJS-12 after THA. Distribution-based approaches and anchor approaches, using WOMAC and OHS as anchors, were used.

## 2. Materials and Methods

This is a quality improvement study. Patients undergoing THA from January 2019 to October 2019 were assessed pre-operatively and six months post-surgery using the FJS-12, the WOMAC and the OHS. The inclusion criteria were severe hip osteoarthritis (Grade III-IV of Kellgren-Lawrence Classification) [20], high and persistent pain, hip replacement surgery and at least 6 months follow-up after surgery. All the patients underwent total hip arthroplasty (anterior and posterior approaches) and were treated by the same surgical equipment. No revisions were performed during the follow-up period.

The exclusion criteria were revision surgery, Grade I-II of Kellgren-Lawrence Classification, simultaneous bilateral hip replacement, hip resurfacing and patients with cognitive impairment. Cognitive impairment could affect the post-operative results, as patients with dementia reported a higher risk of falls and fractures, influencing the outcomes reporting [21].

### 2.1. Assessment Instruments

The FJS-12 [22] consists of 12 questions, with a five-point Likert response format, summed to obtain scores ranging from 12 to 60. The raw score is normalized to range from 0 (worst condition) to 100 points (best condition).

The WOMAC [13] is a clinical orthopedic score used to assess pain, stiffness and physical function in patients. It comprises 24 questions, with a zero to four-point Likert scale response, summed to obtain scores ranging from 0 to 96. The raw score is normalized to range from 0 (worst condition) to 100 points (best condition).

The OHS [23,24] assesses the patient’s perceived pain and functional ability. It comprises 12 questions, with a zero to four-point Likert scale response, summed to obtain scores ranging from 0 to 48.

### 2.2. Statistical Analysis

An a priori power analysis was performed. With an alpha level of 0.05 (two-tailed), minimum power established at 0.80, and a medium effect size of 0.6 (ES, Cohen’s *d*) [25], 24 participants would be necessary to find a statistically significant effect on the model. Data normality for the FJS-12 was assessed using the Kolmogorov-Smirnov test of normality. The baseline and 6 months post-operative scores were compared using the paired sample t-test. The statistical level of significance was set to 0.05. Both distribution-based and anchor approaches were used to estimate MCIDs for the normalized FJS-12 from baseline to 6 months post-operative follow-up.

### 2.3. Distribution-Based Approaches

The distribution-based approaches used: 0.5 standard deviations (05 SD), the standard error of measurement (SEM) and the minimum detectable change (MDC). The 0.5 SD was related to a medium effect size. The SEM represents the smallest change above the measurement error (ME). The MDC represents the smallest change above the measurement error, with a 95% confidence interval. Cronbach’s alpha as the measure of the reliability of the FJS-12 was used to calculating the SEM and MDC.

### 2.4. Anchor Approaches

High values of WOMAC and OHS scores at 6 months follow-up were used as the anchor. The cut-off value for WOMAC was 90 points, obtained from the estimate of the score at 6 months post-operative in the literature [26]; while 40 was set as the cut-off for OHS since the score from 40 to 48 indicate satisfactory joint function [27]. Therefore, patients with WOMAC values greater than 90 and OHS values greater than 40 at 6 months post-operative were considered responders. An external anchor was defined as valid if the correlation coefficient with FJS-12 was at least 0.3 with *p* < 0.05.

Two anchor methods were used: receiver operating characteristic (ROC) curves and the mean change (MC). The ROC curves were used to identify the change in FJS-12 with maximized sensitivity and specificity. To find the cut-off, the Youden Index was used. The MC in FJS-12 is the mean difference in patients who reported high values of WOMAC and OHS scores.

The same anchors were used for estimated PASS. WOMAC and OHS scores as anchors were used to consider pain, physical function and patient satisfaction, as suggested in the literature [28]. PASS thresholds of FJS-12 were calculated, using the 75th percentile of the cumulative percentage curve of patients who consider themselves in an acceptable state of symptoms, and the ROC curve point, which maximized the Youden index. Statistical analyses were performed using SPSS version 26 (IBM Inc., Armonk, NY, USA).

## 3. Results

In this study were included 50 patients, 23 women and 27 men affected by hip osteoarthritis (Grade III-IV of Kellgren-Lawrence Classification). The average age was 73.2 ± 10.5 years. The mean BMI was 28.8 ± 4.7. The outcome questionnaires were completed both pre-operatively and six months post-operatively by the patients.

Normal distribution of FJS-12 at baseline and 6 months was assessed with the Kolmogorov-Smirnov test (*p* = 0.2).

**Figure 1:**
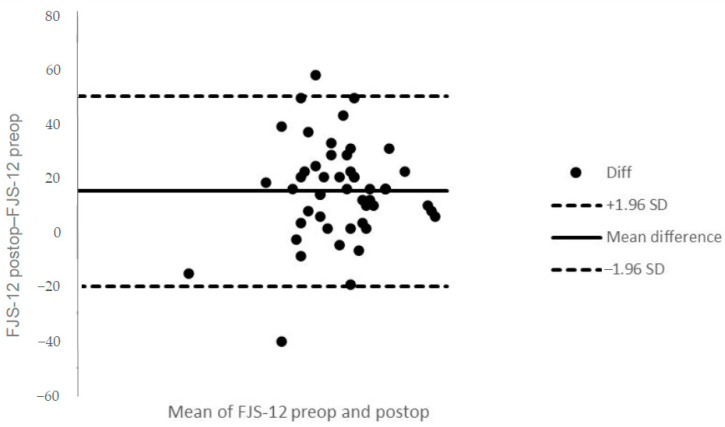
Bland-Altman plot for the mean differences of Forgotten Joint Score 12 (FJS-12) between pre-operative and postoperative follow-up. The mean difference is indicated by the solid horizontal line, and the limits of agreement are demarcated by the dashed horizontal lines.

At the baseline follow-up, the average FJS-12 score was 63.2 ± 14.4, ranging from a minimum of 35.4 to a maximum of 93.8 (0% floor and ceiling effects). At 6 months follow-up, the average FJS-12 score was 78.9 ± 15.6, ranging from a minimum of 22.9 to a maximum of 100 (0% floor effect and 12% ceiling effect). Figure 1 shows the Bland-Altman plot for the mean differences of FJS-12 between pre-operative and post-operative follow-up. A statistically significant change between pre-operative and 6 months follow-ups was found (*p* < 0.001).

The internal consistency reliability for FJS-12 was high (α = 0.8). High correlation between FJS-12 change and WOMAC and OHS at the last follow-up was found (*r* = 0.4, *p* = 0.007; 0.4, *p* = 0.012, respectively). A high correlation between FJS-12 and WOMAC and OHS at the last follow-up was found also (*r* = 0.6, *p* < 0.001; 0.5, *p* < 0.001, respectively). Therefore, WOMAC and OHS were a valid external anchor for both MCID and PASS. 

**Figure 2:**
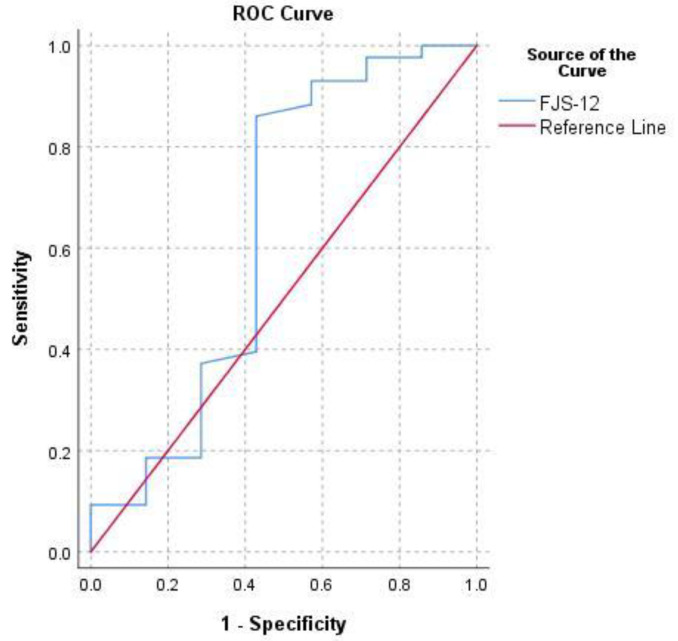
Receiver operating characteristic curve (ROC) for the prediction of FJS-12’s minimum clinically important difference (MCID) based on the value of Western Ontario and McMaster Universities Osteoarthritis Index (WOMAC) ≥90 at last follow-up.

**Figure 3:**
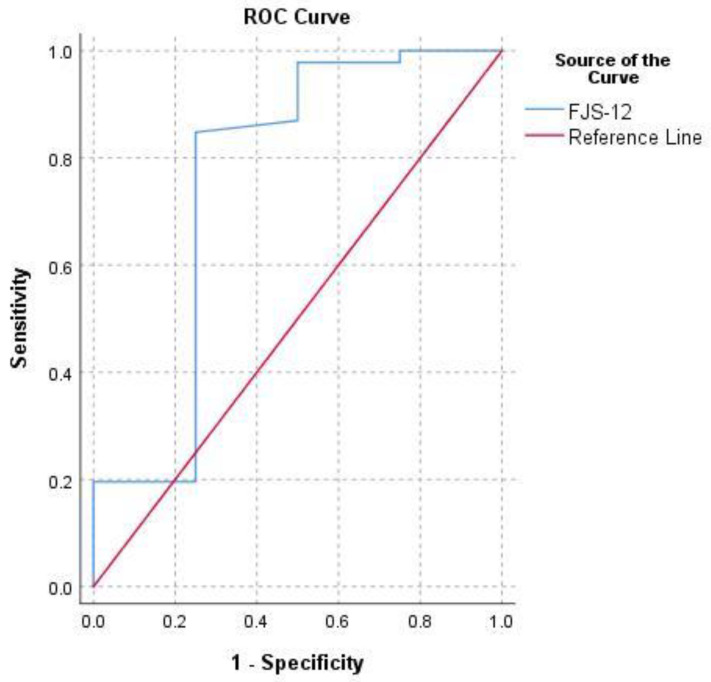
Receiver operating characteristic curve (ROC) for predicting FJS-12’s MCID based on the value of Oxford Hip Score (OHS) ≥40 at last follow-up.

MCID estimates for normalized FJS-12 for hip prosthesis ranged from 3.1 to 21.8. The following MCID was calculated: an MCID of 8.9 (0.5 SD) with a medium effect size (ES = 0.5), an MCID of 7.9 (SEM) with internal consistency reliability of 0.8, an MCID of 21.8 (MDC) at the 95% confidence level, an MCID of 3.1 (ROC) with low-medium instrument responsiveness (AUC = 0.6 for WOMAC and AUC = 0.8 for OHS) (Figure 2 and Figure 3) and an MCID of 17.5 (with WOMAC as an external anchor) and 17.6 (with OHS as an external anchor) Mean Change (MC) (Table 1).

PASS calculated for normalized FJS-12 for hip prosthesis ranged from 69.8 to 91.7. The thresholds of the FJS-12 that maximized the sensitivity and specificity for detecting a PASS were 76 (AUC = 0.7), and 69.8 (AUC = 0.8). The cut-off values computed with the 75th percentile approach were 91.7 and 90.1 (Table 2).

## 4. Discussion

Data from 50 cases who underwent THA were analyzed. The study aimed to find the FJS-12’s MCID and PASS from pre-operative time to 6 months post-operative follow-up in patients who have undergone a THA.

Many studies have analyzed how a score [29] changes between pre- and post-operative and if it reflects the patient’s improvements over time. Bovonratwet et al. reported that patient satisfaction should not be the only proxy for traditional outcome measures of pain relief and functional improvement [10]. Other factors, such as room conditions or inefficient communication with nurses, could decrease the overall satisfaction of the patients after THA [10]. Kurihara et al. reported that other factors beyond surgery such as the early post-operative movements could influence the PROMs [30]. Therefore, a change in values after surgery could be statistically significant, but it may not be considered clinically meaningful by many patients. This paper aimed to define the value of FJS-12, in patients who underwent THA, for whom the change was perceived as beneficial. To answer this question, the FJS-12′s MCID from pre-operative to 6 months post-operative time was calculated.

The term MCID was used for the first time by Jaeschke et al. as “the smallest difference in score in the domain of interest which patients perceive as beneficial” [31]. Therefore, the MCID is a calculated minimum threshold value in a score of interest that patients perceive as a clinical status improvement.

Different methods to compute MCID were reported, divided into distribution-based approaches and anchor approaches. The distribution-based approaches are built upon the statistical properties of a study’s results [32] and include the 0.5 SD method, the SEM and the MDC. The 0.5 SD represents a clinically meaningful change with medium effect size [33]. The SEM is the change due to the unreliability of the scale or measurement errors [34]. A difference smaller than the calculated SEM is probably due to a measurement error rather than a real observed change; therefore, an MCID value smaller than the SEM likely results from error. The MDC is the smallest change that can be considered above the measurement error, with a 95% level of confidence [35]. An MCID value smaller than the MDC was not considered valid. The MCID was determined using the OKS and WOMAC as anchors: the former assessed the difference between the change in the average score of improved and non-improved patients: the latter identified the MCID as the point of the ROC in which sensitivity and specificity are maximized. This cut-off was found with Youden’s Index [36]. This approach can find the best discrimination between responders and non-responders [37]. No agreement has been achieved as to which MCID calculation method is superior. To account for the method and sample-dependent variations, identifying a single threshold that defines the MCID is potentially misleading. Therefore, it is recommended that a plausible range of MCIDs be presented [38]. Consequently, both distribution-based approaches and anchor approaches were used to assess the MCID of FJS-12.

To establish a valid external anchor, the a priori criteria suggested by Revicki et al. was used [39]. Therefore, WOMAC and OHS scores were adopted as external anchors due to their correlation with FJS-12 being higher than 0.3 and statistically significant at the 0.05 level. Patients with WOMAC score ≥90 points, and patients with OHS score ≥40 points at 6 months follow-up were defined as responders [26,27]. The MCIDs calculated with those two different anchors are very close; therefore, the consistency of results across different anchors was confirmed.

The different calculation methods used in this study resulted in different values of MCID. These values ranged from 3.1 to 21.8. The smallest threshold resulted from the ROC method, whereas the greatest threshold was obtained by the MDC method.

Assuming a medium effect size (Cohen’s *d* of 0.5), an MCID of 8.9 was found (0.5 SD). The smallest change that can be considered above the measurement error, with a 95% level of confidence was 20.1 (MDC). The ROC method showed the same MCID both with WOMAC and OHS scores as anchors. Otherwise, this MCID estimated is not valid because it is lower than the estimated SEM. Lastly, the MC method presented an MCID of 17.5 and 17.6, with WOMAC and OHS as the anchor, respectively.

In literature, only one article assessed the MCID of FJS-12 in patients who underwent THA [40]. Although Robinson et al. reported results after one year of follow up (this study has only 6 months of follow up), they used only the 0.5 SD method to assess the MCID. Instead, in this paper, several methods were used for the same purpose. However, the MCID reported by Robinson et al. is very close to those calculated in the present study.

Other authors have estimated the MCID of FJS-12 in patients who underwent different surgical procedures. Ingelsrud et al. reported an MCID of the FJS-12 of 14 points [41] in total knee arthroplasty. Behrend et al. calculated an FJS-12′s MCID of 13 points [42] in anterior cruciate ligament reconstruction. All these MCIDs are also close to those calculated in this paper. Rosinsky et al. [43] determined a threshold for a successful outcome for the FJS-12 after THA. They used a composite score to assess outcomes as an external criterion. The author reported this choice as a limitation of the study due to the lack of a defined tool (as the MCID). Instead, in this paper, two valid and reproducible scores were adopted (MCID and PASS). Moreover, another limitation of the study by Rosinsky et al. [43] was the lack of the pre-operative value of FJS-12. The lack of these data does not allow calculation of the difference between pre- and post-operative outcomes. Therefore, this value was included in the present study.

Copay et al. defined the validity of an MCID with two criteria: “it has to be at least greater than the measurement error, and it has to correspond to the patient perception of the importance of change” [44]. Based on these principles, MC seems to be the more appropriate calculation method for defining an MCID threshold in this study. Firstly, the MCIDs calculated with the MC method were both higher than SEM. Secondly, since the MC is, by definition, the mean change in responders, it corresponds to the patient’s perception of the importance of the change. Furthermore, the FJS-12′s MCIDs calculated with the MC method are closer to the FJS-12′s MCID calculated by Robinson et al. for patients who underwent THA. Methods 0.5 SD and MDC were also higher than SEM, but anchoring approaches are most commonly used in newer orthopedic studies [13,15,45]. Since the values calculated with the two different anchors (WOMAC and OHS) are very similar, only one was considered as the MCID. WOMAC has a higher correlation with the FJS-12 than the OHS and considers more dimensions than the OHS. Therefore, WOMAC was used as an anchor to calculate the MCID. Based on these premises, the FJS-12′s MCID is 17.5 for patients who underwent THA.

The second purpose of the present study was to evaluate the PASS of FJS-12 at 6 months after THA. PASS is the threshold on a PROM most closely associated with patient satisfaction, assessed on a separate questionnaire [14]. The most used approaches to calculate the PASS are the 75th percentile approach and the ROC curves [28]. The first identifies the cut-off point corresponding to the 75th percentile of the scores in patients who report a satisfactory health status by the anchoring question. The ROC method finds the threshold that is the best compromise between sensitivity and specificity for each outcome criterion, with Youden’s Index. Both methods described above were used, calculating different values of PASS. The thresholds ranged from 69.8 to 91.7. The smallest threshold resulted from the ROC method, whereas the 75th percentile approach was the greatest threshold. The ROC method showed the following cut-offs: 76 (AUC = 0.7) with WOMAC as anchor and 69.8 (AUC = 0.8) with OHS as the anchor. The 75th percentile approach showed a cut-off of 91.7 with WOMAC as anchor and 90.1 with OHS as the anchor.

In the literature, only one article reported the PASS of FJS-12 in patients who underwent THA [46]. In this study, Galea et al. reported the FJS-12′s PASS at 3 months, and 1-year and 2-year follow-up. Instead, in this paper, the follow-up was shorter (6 months). Compared with the literature, the most reasonable PASS value calculated was assessed by the ROC method. Furthermore, this threshold has the highest AUC value.

This study has different points of strength. To our knowledge, this was the first study that calculated the MCID of FJS-12 in patients who underwent THA using anchor-approaches methods. Secondly, the questionnaires used as anchors are valid and commonly adopted in the literature (WOMAC and OHS). Moreover, since two anchors (i.e., the high value of WOMAC and OHS) for MCID and PASS were accessible to be used, the consistency of results across different anchors was assessed. Lastly, the most common ad hoc methods were used to calculate the MCID and PASS.

However, this paper also has limitations. The MCID and PASS were calculated for 6 months follow-up and cannot deliver information about longer-term FJS-12 scores. The MCID and PASS values may be different depending on the follow-up time. Moreover, even if the sample size was sufficient according to the power analysis, a higher number of patients were used in the literature. Lastly, the population of the present study included THA performed by anterior and posterior approaches. Although the heterogeneity could influence the results, this paper aimed to determine a value for outcome improvement based on patients’ perceptions after surgery, regardless of the approach adopted during surgery.

## 5. Conclusions

The FJS-2′s MCID and PASS in patients who underwent THA from baseline to 6 months post-operative follow-up were calculated. Both distribution-based and anchor approaches to calculate MCID were adopted. The MCID of FJS-12 is 17.5. Patients who reported this change in the FJS-12 score achieved a clinically significant improvement after THA, from baseline to 6-month post-operative follow-up. The FJS-12′s PASS is 69.8; therefore, an FJS-12 value at least of 69.8 at 6-month follow-up indicates patient satisfaction about the symptoms.

## Figures and Tables

**Table 1:** MCID for FJS-12 calculated by both distribution-based and anchor approaches.

MCID	Cut-Off Value	Anchor
0.5 SD	8.9	/
SEM	7.9	/
MDC	21.8	/
ROC (AUC)	3.1 (0.6)	WOMAC
ROC (AUC)	3.1 (0.8)	OHS
MC	17.5	WOMAC
MC	17.6	OHS

AUC: area under the curve; MC: mean change; MCID: minimum clinically important difference; MDC: minimum detectable change; OHS: Oxford Hip Score; ROC: receiver operating characteristic; SEM: standard error of measurement; WOMAC: Western Ontario and McMaster Universities Osteoarthritis Index.

**Table 2:** Patient acceptable symptom state (PASS) for FJS-12 calculated by both distribution-based and anchor approaches.

PASS	Cut-Off Value	Anchor
ROC (AUC)	76 (0.7)	WOMAC
ROC (AUC)	69.8 (0.8)	OHS
75th percentile	91.7	WOMAC
75th percentile	90.1	OHS

AUC: area under the curve; OHS: Oxford Hip Score; WOMAC: Western Ontario and McMaster Universities Osteoarthritis Index.

## Data Availability

The data presented in this study are available on request from the corresponding author. The data are not publicly available due to privacy.

## Abbreviations

05 SD—0.5 standard deviation

FJS-12—Forgotten Joint Score-12

MC—mean change

MCID—minimum clinically important difference

MDC—minimum detectable change

MIC—minimum important change

OHS—the Oxford Hip Score

PASS—patient acceptable symptom state

PROMs—patient-reported outcome measures

ROC/AUC—receiver operating characteristic/area under the curve

SEM—standard error of measurement

SF-36—Short Form Health Survey

THA—total hip arthroplasty

WOMAC—Western Ontario and McMaster Universities Osteoarthritis Index
